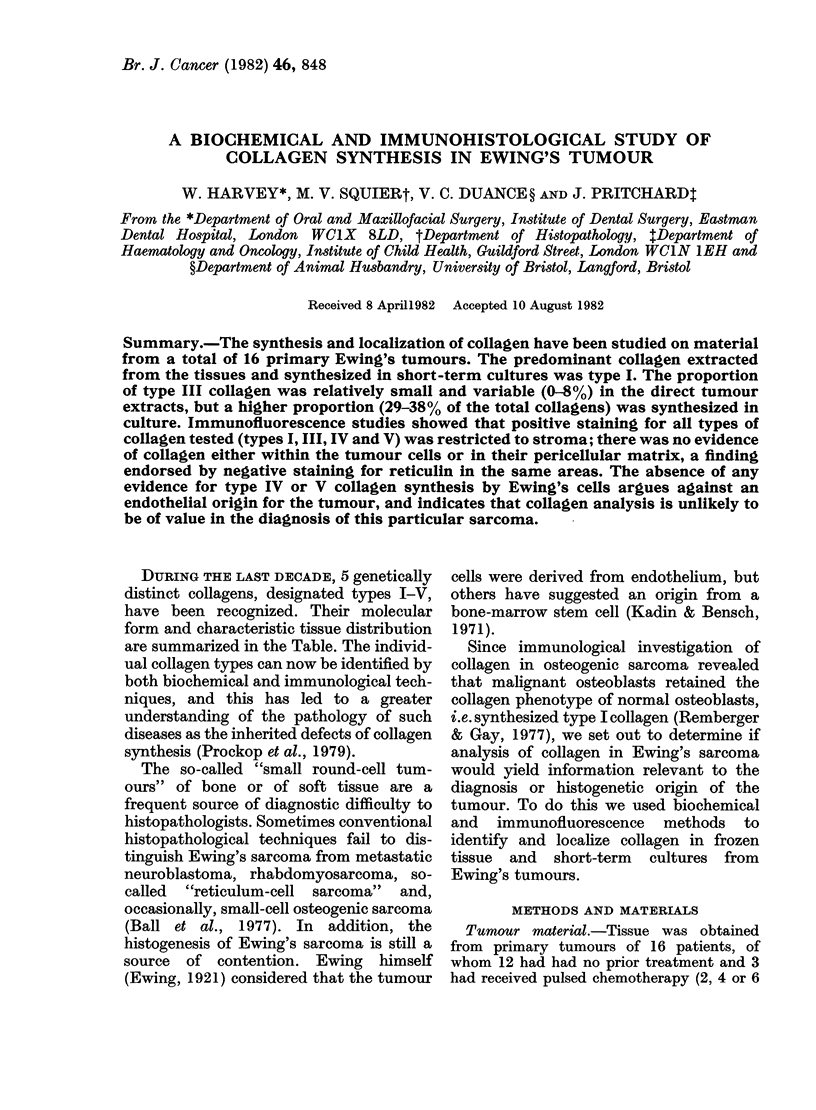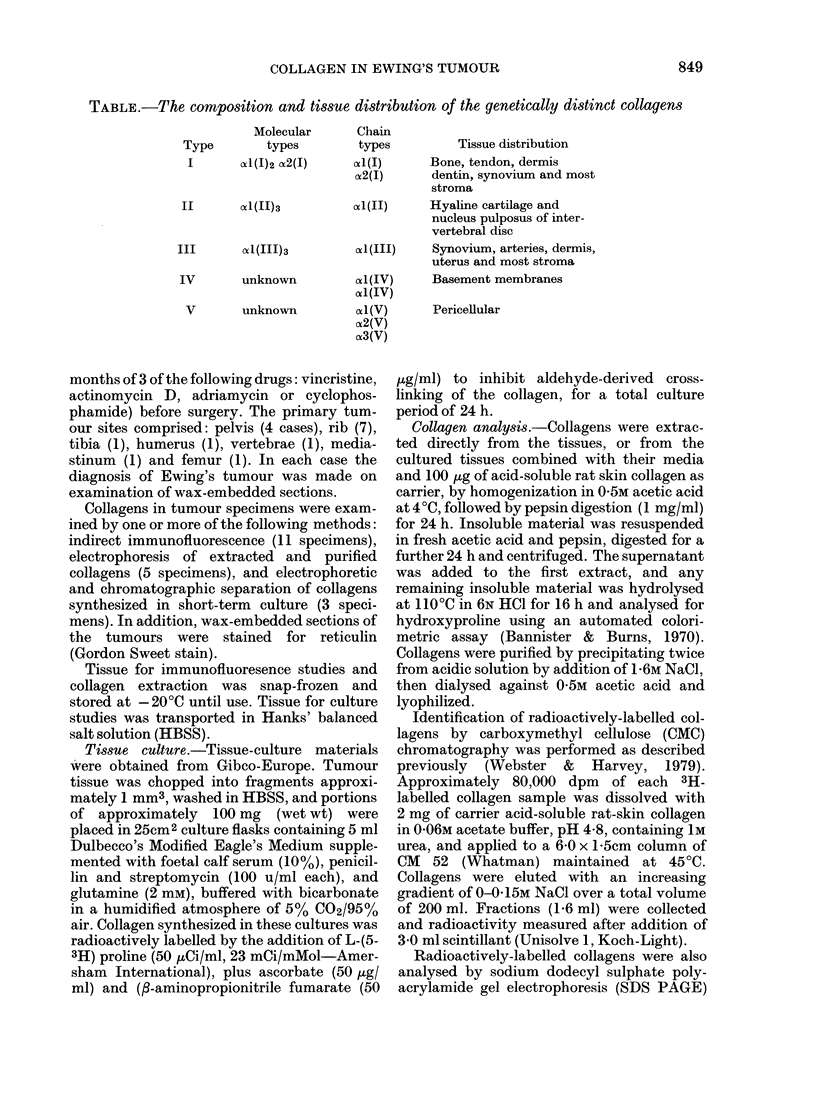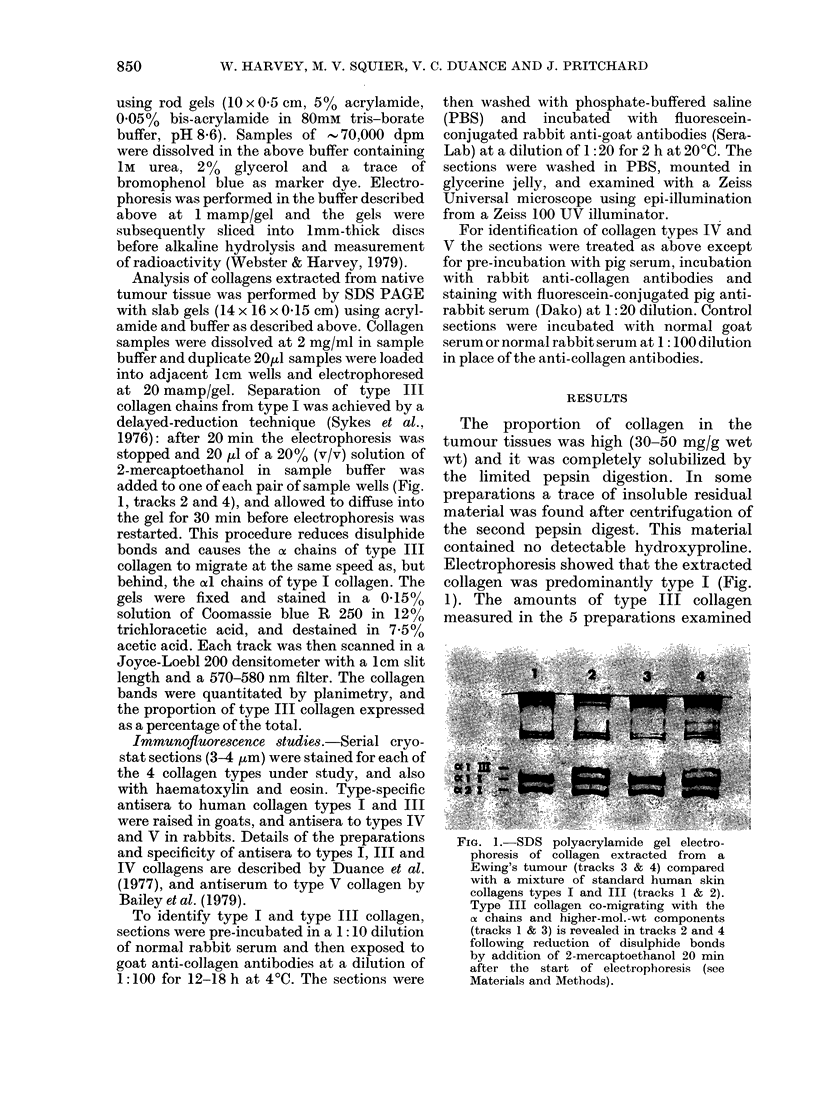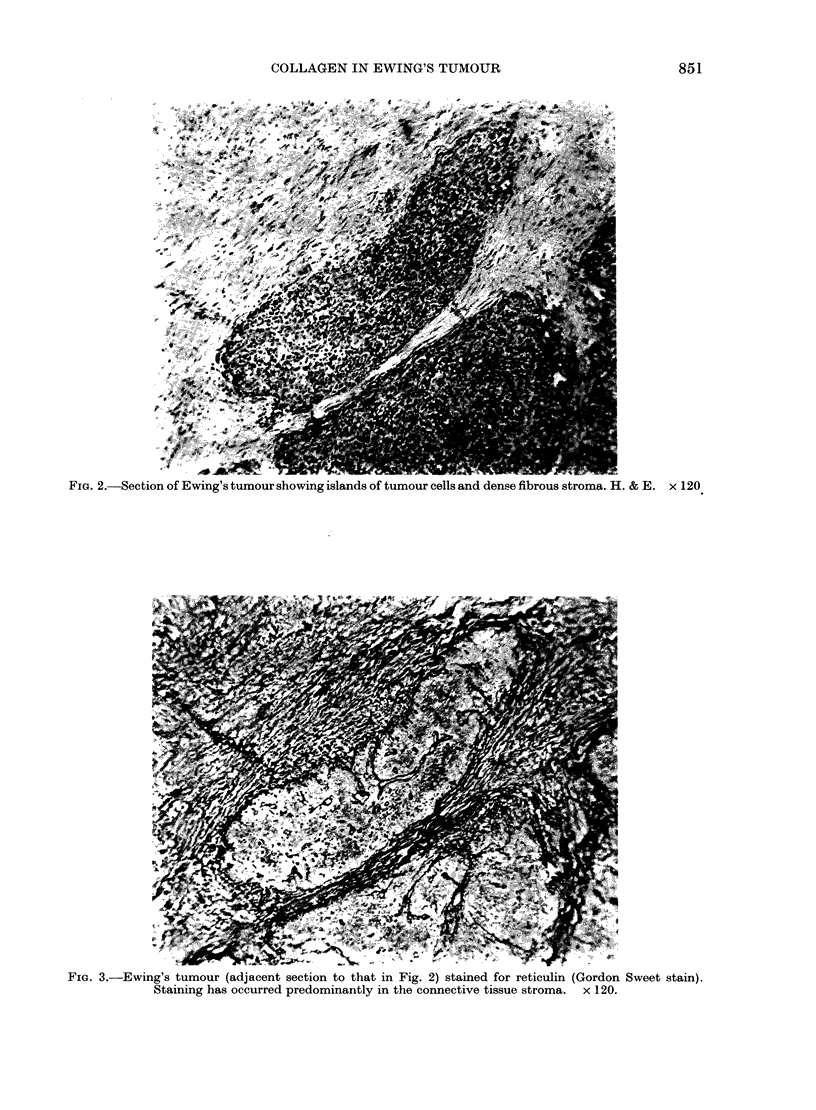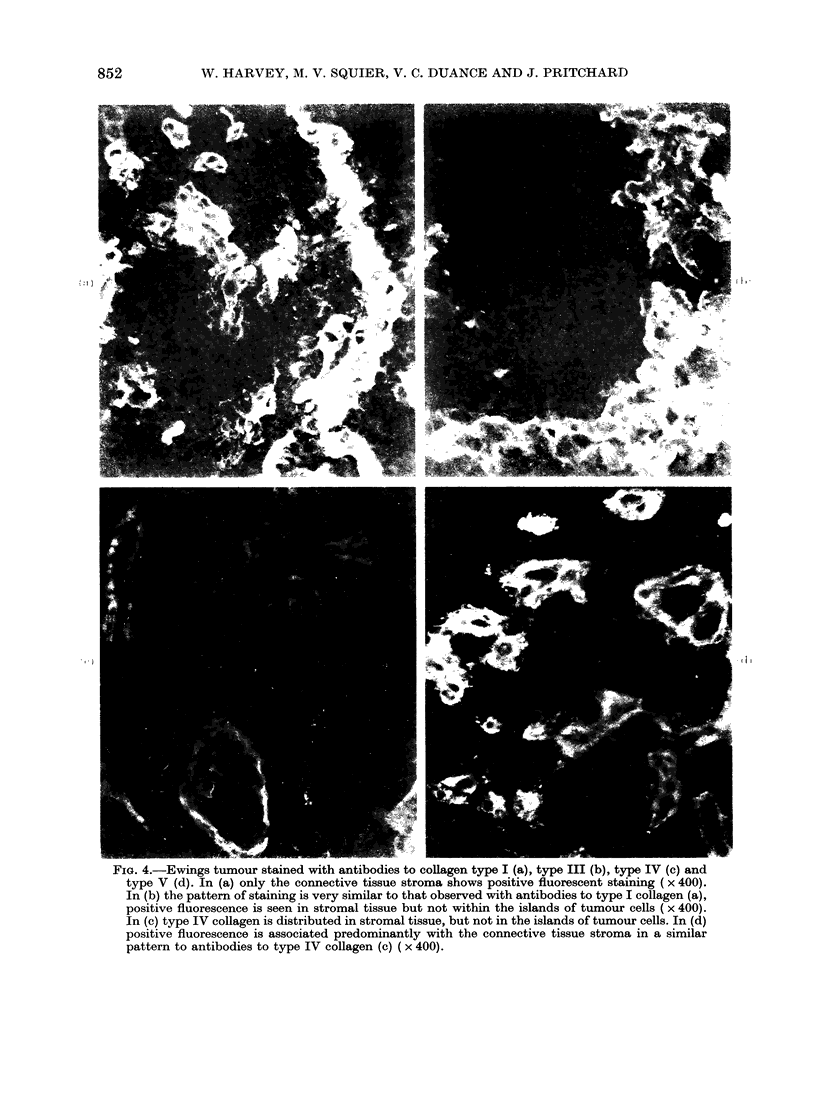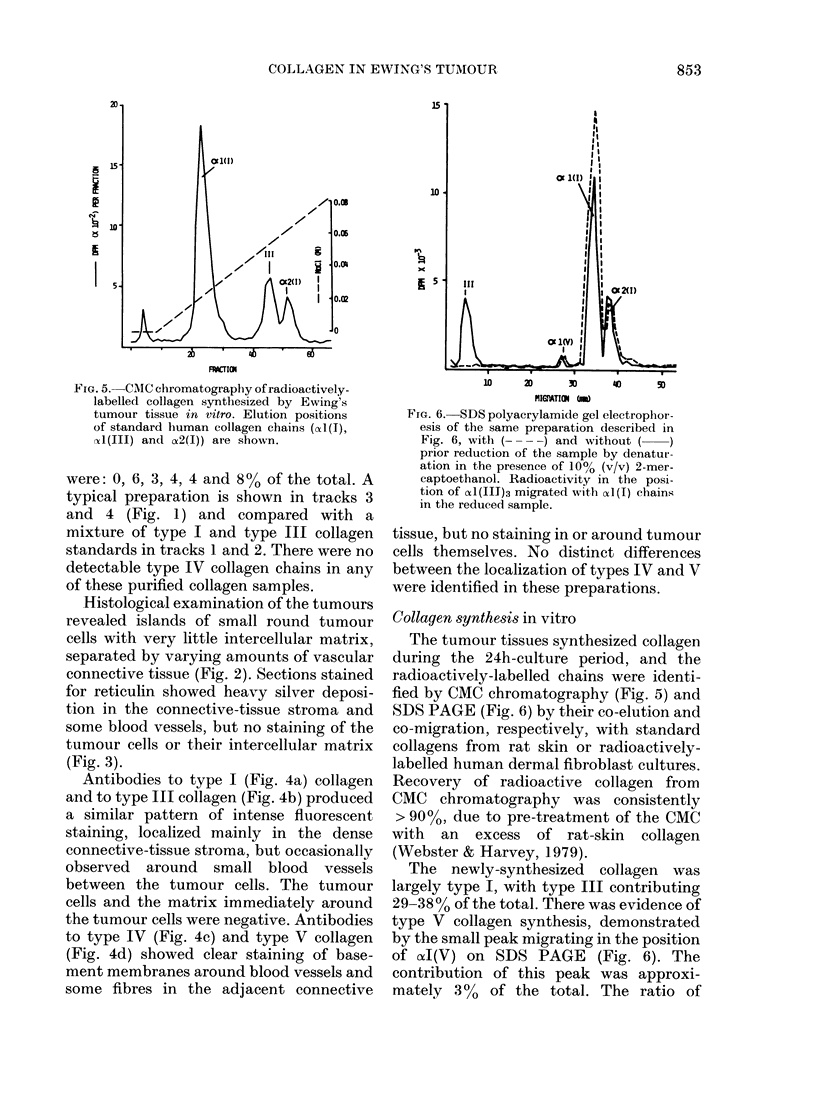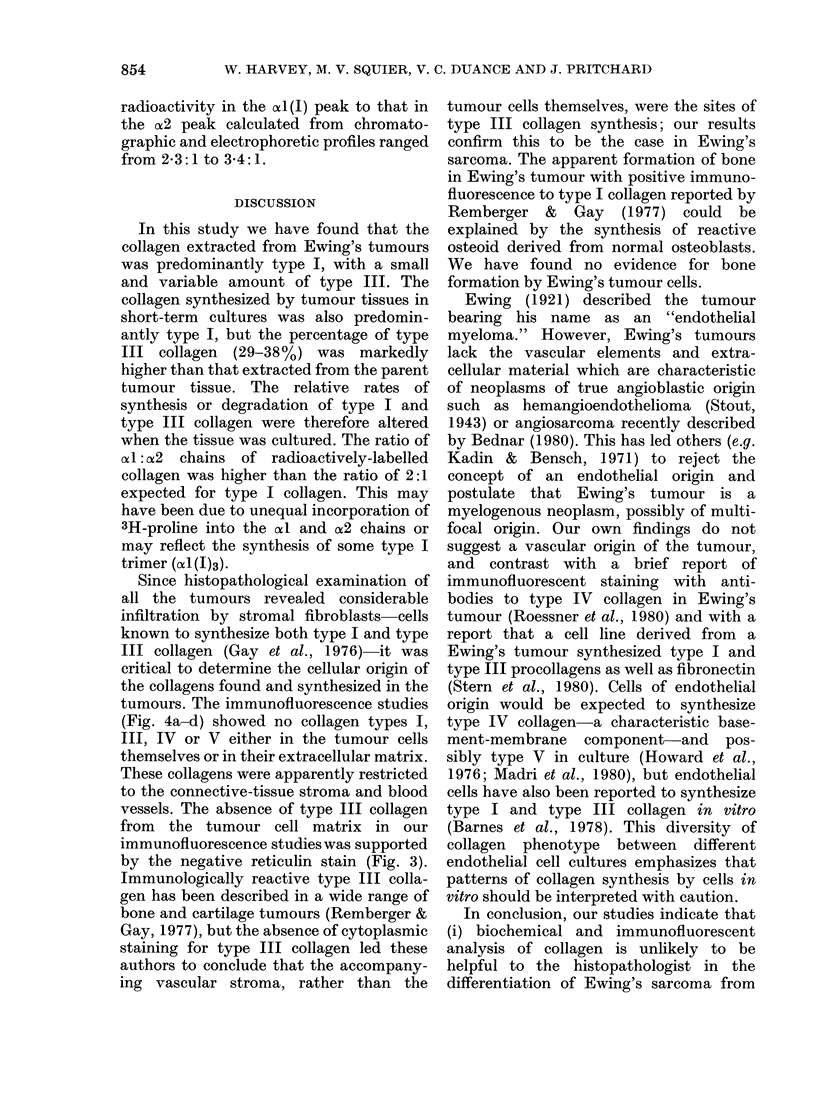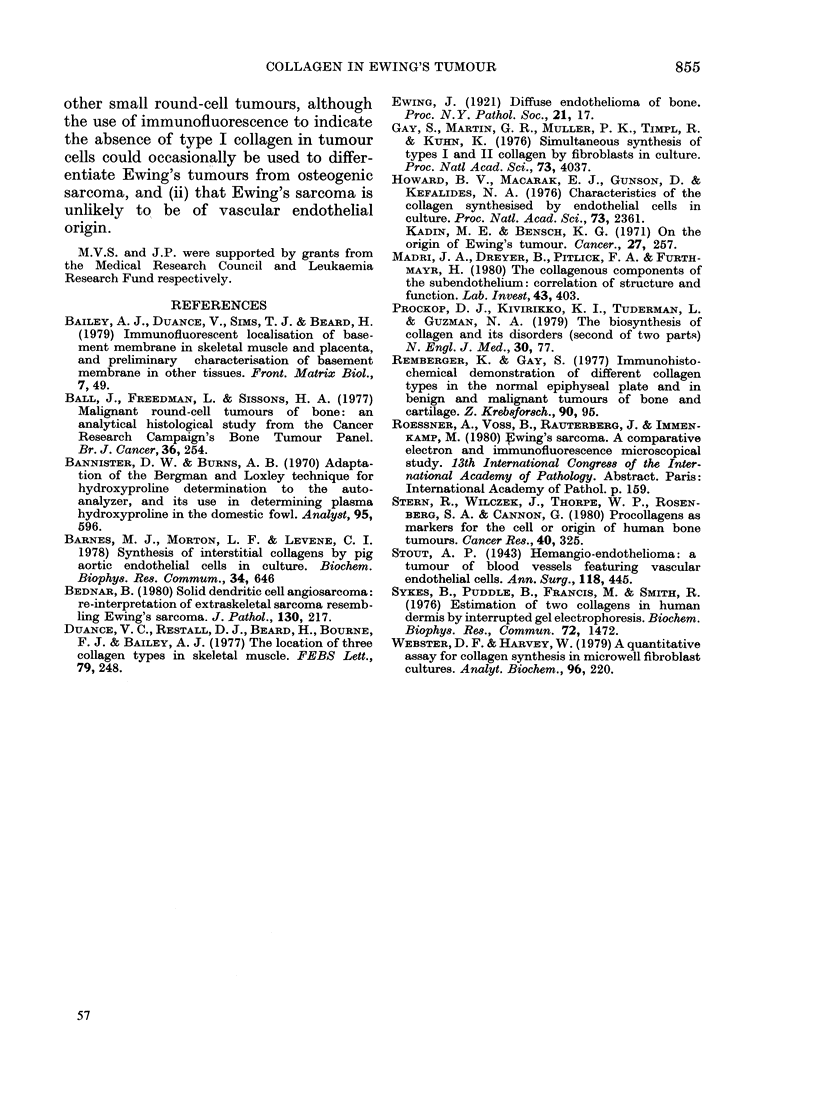# A biochemical and immunohistological study of collagen synthesis in Ewing's tumour.

**DOI:** 10.1038/bjc.1982.294

**Published:** 1982-12

**Authors:** W. Harvey, M. V. Squier, V. C. Duance, J. Pritchard

## Abstract

**Images:**


*Br. J. Cancer* (1982) **46**, 848

### A BIOCHEMICAL AND IMMUNOHISTOLOGICAL STUDY OF COLLAGEN SYNTHESIS IN EWING'S TUMOUR

W. HARVEY*, M. V. SQUIER†, V. C. DUANCE§ AND J. PRITCHARD‡

*From the *Department of Oral and Maxillofacial Surgery, Institute of Dental Surgery, Eastman Dental Hospital, London WC1X 8LD, †Department of Histopathology, ‡Department of Haematology and Oncology, Institute of Child Health, Guildford Street, London WC1N 1EH and §Department of Animal Husbandry, University of Bristol, Langford, Bristol*

Received 8 April1982 Accepted 10 August 1982

**Summary.—The synthesis and localization of collagen have been studied on material from a total of 16 primary Ewing's tumours. The predominant collagen extracted from the tissues and synthesized in short-term cultures was type I. The proportion of type III collagen was relatively small and variable (0–8%) in the direct tumour extracts, but a higher proportion (29–38% of the total collagens) was synthesized in culture. Immunofluorescence studies showed that positive staining for all types of collagen tested (types I, III, IV and V) was restricted to stroma; there was no evidence of collagen either within the tumour cells or in their pericellular matrix, a finding endorsed by negative staining for reticulin in the same areas. The absence of any evidence for type IV or V collagen synthesis by Ewing's cells argues against an endothelial origin for the tumour, and indicates that collagen analysis is unlikely to be of value in the diagnosis of this particular sarcoma.**

DURING THE LAST DECADE, 5 genetically distinct collagens, designated types I–V, have been recognized. Their molecular form and characteristic tissue distribution are summarized in the Table. The individual collagen types can now be identified by both biochemical and immunological techniques, and this has led to a greater understanding of the pathology of such diseases as the inherited defects of collagen synthesis (Prockop *et al.*, 1979).

The so-called "small round-cell tumours" of bone or of soft tissue are a frequent source of diagnostic difficulty to histopathologists. Sometimes conventional histopathological techniques fail to distinguish Ewing's sarcoma from metastatic neuroblastoma, rhabdomyosarcoma, so-called "reticulum-cell sarcoma" and, occasionally, small-cell osteogenic sarcoma (Ball *et al.*, 1977). In addition, the histogenesis of Ewing's sarcoma is still a source of contention. Ewing himself (Ewing, 1921) considered that the tumour cells were derived from endothelium, but others have suggested an origin from a bone-marrow stem cell (Kadin & Bensch, 1971).

Since immunological investigation of collagen in osteogenic sarcoma revealed that malignant osteoblasts retained the collagen phenotype of normal osteoblasts, *i.e.* synthesized type I collagen (Remberger & Gay, 1977), we set out to determine if analysis of collagen in Ewing's sarcoma would yield information relevant to the diagnosis or histogenetic origin of the tumour. To do this we used biochemical and immunofluorescence methods to identify and localize collagen in frozen tissue and short-term cultures from Ewing's tumours.

### METHODS AND MATERIALS

*Tumour material.*—Tissue was obtained from primary tumours of 16 patients, of whom 12 had had no prior treatment and 3 had received pulsed chemotherapy (2, 4 or 6

TABLE.—*The composition and tissue distribution of the genetically distinct collagens*

| Type | Molecular types | Chain types | Tissue distribution |
|---|---|---|---|
| I | $\alpha 1(I)_2\ \alpha 2(I)$ | $\alpha 1(I)$<br>$\alpha 2(I)$ | Bone, tendon, dermis dentin, synovium and most stroma |
| II | $\alpha 1(II)_3$ | $\alpha 1(II)$ | Hyaline cartilage and nucleus pulposus of intervertebral disc |
| III | $\alpha 1(III)_3$ | $\alpha 1(III)$ | Synovium, arteries, dermis, uterus and most stroma |
| IV | unknown | $\alpha 1(IV)$<br>$\alpha 1(IV)$ | Basement membranes |
| V | unknown | $\alpha 1(V)$<br>$\alpha 2(V)$<br>$\alpha 3(V)$ | Pericellular |

months of 3 of the following drugs: vincristine, actinomycin D, adriamycin or cyclophosphamide) before surgery. The primary tumour sites comprised: pelvis (4 cases), rib (7), tibia (1), humerus (1), vertebrae (1), mediastinum (1) and femur (1). In each case the diagnosis of Ewing's tumour was made on examination of wax-embedded sections.

Collagens in tumour specimens were examined by one or more of the following methods: indirect immunofluorescence (11 specimens), electrophoresis of extracted and purified collagens (5 specimens), and electrophoretic and chromatographic separation of collagens synthesized in short-term culture (3 specimens). In addition, wax-embedded sections of the tumours were stained for reticulin (Gordon Sweet stain).

Tissue for immunofluoresence studies and collagen extraction was snap-frozen and stored at $-20\,°C$ until use. Tissue for culture studies was transported in Hanks' balanced salt solution (HBSS).

*Tissue culture.*—Tissue-culture materials were obtained from Gibco-Europe. Tumour tissue was chopped into fragments approximately 1 $mm^3$, washed in HBSS, and portions of approximately 100 mg (wet wt) were placed in 25$cm^2$ culture flasks containing 5 ml Dulbecco's Modified Eagle's Medium supplemented with foetal calf serum (10%), penicillin and streptomycin (100 u/ml each), and glutamine (2 mM), buffered with bicarbonate in a humidified atmosphere of 5% $CO_2$/95% air. Collagen synthesized in these cultures was radioactively labelled by the addition of L-(5-$^3H$) proline (50 $\mu$Ci/ml, 23 mCi/mMol—Amersham International), plus ascorbate (50 $\mu$g/ml) and ($\beta$-aminopropionitrile fumarate (50 $\mu$g/ml) to inhibit aldehyde-derived cross-linking of the collagen, for a total culture period of 24 h.

*Collagen analysis.*—Collagens were extracted directly from the tissues, or from the cultured tissues combined with their media and 100 $\mu$g of acid-soluble rat skin collagen as carrier, by homogenization in 0·5M acetic acid at 4 °C, followed by pepsin digestion (1 mg/ml) for 24 h. Insoluble material was resuspended in fresh acetic acid and pepsin, digested for a further 24 h and centrifuged. The supernatant was added to the first extract, and any remaining insoluble material was hydrolysed at 110°C in 6N HCl for 16 h and analysed for hydroxyproline using an automated colorimetric assay (Bannister & Burns, 1970). Collagens were purified by precipitating twice from acidic solution by addition of 1·6M NaCl, then dialysed against 0·5M acetic acid and lyophilized.

Identification of radioactively-labelled collagens by carboxymethyl cellulose (CMC) chromatography was performed as described previously (Webster & Harvey, 1979). Approximately 80,000 dpm of each $^3H$-labelled collagen sample was dissolved with 2 mg of carrier acid-soluble rat-skin collagen in 0·06M acetate buffer, pH 4·8, containing 1M urea, and applied to a 6·0 × 1·5cm column of CM 52 (Whatman) maintained at 45°C. Collagens were eluted with an increasing gradient of 0–0·15M NaCl over a total volume of 200 ml. Fractions (1·6 ml) were collected and radioactivity measured after addition of 3·0 ml scintillant (Unisolve 1, Koch-Light).

Radioactively-labelled collagens were also analysed by sodium dodecyl sulphate polyacrylamide gel electrophoresis (SDS PAGE)

using rod gels ($10 \times 0{\cdot}5$ cm, 5% acrylamide, 0·05% bis-acrylamide in 80mM tris–borate buffer, pH 8·6). Samples of ~70,000 dpm were dissolved in the above buffer containing 1M urea, 2% glycerol and a trace of bromophenol blue as marker dye. Electrophoresis was performed in the buffer described above at 1 mamp/gel and the gels were subsequently sliced into 1mm-thick discs before alkaline hydrolysis and measurement of radioactivity (Webster & Harvey, 1979).

Analysis of collagens extracted from native tumour tissue was performed by SDS PAGE with slab gels ($14 \times 16 \times 0{\cdot}15$ cm) using acrylamide and buffer as described above. Collagen samples were dissolved at 2 mg/ml in sample buffer and duplicate 20$\mu$l samples were loaded into adjacent 1cm wells and electrophoresed at 20 mamp/gel. Separation of type III collagen chains from type I was achieved by a delayed-reduction technique (Sykes *et al.*, 1976): after 20 min the electrophoresis was stopped and 20 $\mu$l of a 20% (v/v) solution of 2-mercaptoethanol in sample buffer was added to one of each pair of sample wells (Fig. 1, tracks 2 and 4), and allowed to diffuse into the gel for 30 min before electrophoresis was restarted. This procedure reduces disulphide bonds and causes the $\alpha$ chains of type III collagen to migrate at the same speed as, but behind, the $\alpha$1 chains of type I collagen. The gels were fixed and stained in a 0·15% solution of Coomassie blue R 250 in 12% trichloracetic acid, and destained in 7·5% acetic acid. Each track was then scanned in a Joyce-Loebl 200 densitometer with a 1cm slit length and a 570–580 nm filter. The collagen bands were quantitated by planimetry, and the proportion of type III collagen expressed as a percentage of the total.

*Immunofluorescence studies.*—Serial cryostat sections (3–4 $\mu$m) were stained for each of the 4 collagen types under study, and also with haematoxylin and eosin. Type-specific antisera to human collagen types I and III were raised in goats, and antisera to types IV and V in rabbits. Details of the preparations and specificity of antisera to types I, III and IV collagens are described by Duance *et al.* (1977), and antiserum to type V collagen by Bailey *et al.* (1979).

To identify type I and type III collagen, sections were pre-incubated in a 1:10 dilution of normal rabbit serum and then exposed to goat anti-collagen antibodies at a dilution of 1:100 for 12–18 h at 4°C. The sections were then washed with phosphate-buffered saline (PBS) and incubated with fluorescein-conjugated rabbit anti-goat antibodies (Sera-Lab) at a dilution of 1:20 for 2 h at 20°C. The sections were washed in PBS, mounted in glycerine jelly, and examined with a Zeiss Universal microscope using epi-illumination from a Zeiss 100 UV illuminator.

For identification of collagen types IV and V the sections were treated as above except for pre-incubation with pig serum, incubation with rabbit anti-collagen antibodies and staining with fluorescein-conjugated pig anti-rabbit serum (Dako) at 1:20 dilution. Control sections were incubated with normal goat serum or normal rabbit serum at 1:100 dilution in place of the anti-collagen antibodies.

### RESULTS

The proportion of collagen in the tumour tissues was high (30–50 mg/g wet wt) and it was completely solubilized by the limited pepsin digestion. In some preparations a trace of insoluble residual material was found after centrifugation of the second pepsin digest. This material contained no detectable hydroxyproline. Electrophoresis showed that the extracted collagen was predominantly type I (Fig. 1). The amounts of type III collagen measured in the 5 preparations examined

FIG. 1.—SDS polyacrylamide gel electrophoresis of collagen extracted from a Ewing's tumour (tracks 3 & 4) compared with a mixture of standard human skin collagens types I and III (tracks 1 & 2). Type III collagen co-migrating with the $\alpha$ chains and higher-mol.-wt components (tracks 1 & 3) is revealed in tracks 2 and 4 following reduction of disulphide bonds by addition of 2-mercaptoethanol 20 min after the start of electrophoresis (see Materials and Methods).

FIG. 2.—Section of Ewing's tumour showing islands of tumour cells and dense fibrous stroma. H. & E. × 120.

FIG. 3.—Ewing's tumour (adjacent section to that in Fig. 2) stained for reticulin (Gordon Sweet stain). Staining has occurred predominantly in the connective tissue stroma. × 120.

Fig. 5.—CMC chromatography of radioactively-labelled collagen synthesized by Ewing's tumour tissue *in vitro*. Elution positions of standard human collagen chains (α1(I), α1(III) and α2(I)) are shown.

Fig. 6.—SDS polyacrylamide gel electrophoresis of the same preparation described in Fig. 6, with (– – – –) and without (———) prior reduction of the sample by denaturation in the presence of 10% (v/v) 2-mercaptoethanol. Radioactivity in the position of α1(III)$_3$ migrated with α1(I) chains in the reduced sample.

were: 0, 6, 3, 4, 4 and 8% of the total. A typical preparation is shown in tracks 3 and 4 (Fig. 1) and compared with a mixture of type I and type III collagen standards in tracks 1 and 2. There were no detectable type IV collagen chains in any of these purified collagen samples.

Histological examination of the tumours revealed islands of small round tumour cells with very little intercellular matrix, separated by varying amounts of vascular connective tissue (Fig. 2). Sections stained for reticulin showed heavy silver deposition in the connective-tissue stroma and some blood vessels, but no staining of the tumour cells or their intercellular matrix (Fig. 3).

Antibodies to type I (Fig. 4a) collagen and to type III collagen (Fig. 4b) produced a similar pattern of intense fluorescent staining, localized mainly in the dense connective-tissue stroma, but occasionally observed around small blood vessels between the tumour cells. The tumour cells and the matrix immediately around the tumour cells were negative. Antibodies to type IV (Fig. 4c) and type V collagen (Fig. 4d) showed clear staining of basement membranes around blood vessels and some fibres in the adjacent connective tissue, but no staining in or around tumour cells themselves. No distinct differences between the localization of types IV and V were identified in these preparations.

### *Collagen synthesis* in vitro

The tumour tissues synthesized collagen during the 24h-culture period, and the radioactively-labelled chains were identified by CMC chromatography (Fig. 5) and SDS PAGE (Fig. 6) by their co-elution and co-migration, respectively, with standard collagens from rat skin or radioactively-labelled human dermal fibroblast cultures. Recovery of radioactive collagen from CMC chromatography was consistently > 90%, due to pre-treatment of the CMC with an excess of rat-skin collagen (Webster & Harvey, 1979).

The newly-synthesized collagen was largely type I, with type III contributing 29–38% of the total. There was evidence of type V collagen synthesis, demonstrated by the small peak migrating in the position of αI(V) on SDS PAGE (Fig. 6). The contribution of this peak was approximately 3% of the total. The ratio of radioactivity in the $\alpha 1(I)$ peak to that in the $\alpha 2$ peak calculated from chromatographic and electrophoretic profiles ranged from 2·3:1 to 3·4:1.

### DISCUSSION

In this study we have found that the collagen extracted from Ewing's tumours was predominantly type I, with a small and variable amount of type III. The collagen synthesized by tumour tissues in short-term cultures was also predominantly type I, but the percentage of type III collagen (29–38%) was markedly higher than that extracted from the parent tumour tissue. The relative rates of synthesis or degradation of type I and type III collagen were therefore altered when the tissue was cultured. The ratio of $\alpha 1:\alpha 2$ chains of radioactively-labelled collagen was higher than the ratio of 2:1 expected for type I collagen. This may have been due to unequal incorporation of $^{3}H$-proline into the $\alpha 1$ and $\alpha 2$ chains or may reflect the synthesis of some type I trimer ($\alpha 1(I)_3$).

Since histopathological examination of all the tumours revealed considerable infiltration by stromal fibroblasts—cells known to synthesize both type I and type III collagen (Gay *et al.*, 1976)—it was critical to determine the cellular origin of the collagens found and synthesized in the tumours. The immunofluorescence studies (Fig. 4a–d) showed no collagen types I, III, IV or V either in the tumour cells themselves or in their extracellular matrix. These collagens were apparently restricted to the connective-tissue stroma and blood vessels. The absence of type III collagen from the tumour cell matrix in our immunofluorescence studies was supported by the negative reticulin stain (Fig. 3). Immunologically reactive type III collagen has been described in a wide range of bone and cartilage tumours (Remberger & Gay, 1977), but the absence of cytoplasmic staining for type III collagen led these authors to conclude that the accompanying vascular stroma, rather than the tumour cells themselves, were the sites of type III collagen synthesis; our results confirm this to be the case in Ewing's sarcoma. The apparent formation of bone in Ewing's tumour with positive immunofluorescence to type I collagen reported by Remberger & Gay (1977) could be explained by the synthesis of reactive osteoid derived from normal osteoblasts. We have found no evidence for bone formation by Ewing's tumour cells.

Ewing (1921) described the tumour bearing his name as an "endothelial myeloma." However, Ewing's tumours lack the vascular elements and extracellular material which are characteristic of neoplasms of true angioblastic origin such as hemangioendothelioma (Stout, 1943) or angiosarcoma recently described by Bednar (1980). This has led others (*e.g.* Kadin & Bensch, 1971) to reject the concept of an endothelial origin and postulate that Ewing's tumour is a myelogenous neoplasm, possibly of multifocal origin. Our own findings do not suggest a vascular origin of the tumour, and contrast with a brief report of immunofluorescent staining with antibodies to type IV collagen in Ewing's tumour (Roessner *et al.*, 1980) and with a report that a cell line derived from a Ewing's tumour synthesized type I and type III procollagens as well as fibronectin (Stern *et al.*, 1980). Cells of endothelial origin would be expected to synthesize type IV collagen—a characteristic basement-membrane component—and possibly type V in culture (Howard *et al.*, 1976; Madri *et al.*, 1980), but endothelial cells have also been reported to synthesize type I and type III collagen *in vitro* (Barnes *et al.*, 1978). This diversity of collagen phenotype between different endothelial cell cultures emphasizes that patterns of collagen synthesis by cells *in vitro* should be interpreted with caution.

In conclusion, our studies indicate that (i) biochemical and immunofluorescent analysis of collagen is unlikely to be helpful to the histopathologist in the differentiation of Ewing's sarcoma from other small round-cell tumours, although the use of immunofluorescence to indicate the absence of type I collagen in tumour cells could occasionally be used to differentiate Ewing's tumours from osteogenic sarcoma, and (ii) that Ewing's sarcoma is unlikely to be of vascular endothelial origin.

M.V.S. and J.P. were supported by grants from the Medical Research Council and Leukaemia Research Fund respectively.

### REFERENCES

BAILEY, A. J., DUANCE, V., SIMS, T. J. & BEARD, H. (1979) Immunofluorescent localisation of basement membrane in skeletal muscle and placenta, and preliminary characterisation of basement membrane in other tissues. *Front. Matrix Biol.*, **7**, 49.

BALL, J., FREEDMAN, L. & SISSONS, H. A. (1977) Malignant round-cell tumours of bone: an analytical histological study from the Cancer Research Campaign's Bone Tumour Panel. *Br. J. Cancer*, **36**, 254.

BANNISTER, D. W. & BURNS, A. B. (1970) Adaptation of the Bergman and Loxley technique for hydroxyproline determination to the autoanalyzer, and its use in determining plasma hydroxyproline in the domestic fowl. *Analyst*, **95**, 596.

BARNES, M. J., MORTON, L. F. & LEVENE, C. I. 1978) Synthesis of interstitial collagens by pig aortic endothelial cells in culture. *Biochem. Biophys. Res. Commum.*, **34**, 646

BEDNAR, B. (1980) Solid dendritic cell angiosarcoma: re-interpretation of extraskeletal sarcoma resembling Ewing's sarcoma. *J. Pathol.*, **130**, 217.

DUANCE, V. C., RESTALL, D. J., BEARD, H., BOURNE, F. J. & BAILEY, A. J. (1977) The location of three collagen types in skeletal muscle. *FEBS Lett.*, **79**, 248.

EWING, J. (1921) Diffuse endothelioma of bone. *Proc. N.Y. Pathol. Soc.*, **21**, 17.

GAY, S., MARTIN, G. R., MULLER, P. K., TIMPL, R. & KUHN, K. (1976) Simultaneous synthesis of types I and II collagen by fibroblasts in culture. *Proc. Natl Acad. Sci.*, **73**, 4037.

HOWARD, B. V., MACARAK, E. J., GUNSON, D. & KEFALIDES, N. A. (1976) Characteristics of the collagen synthesised by endothelial cells in culture. *Proc. Natl. Acad. Sci.*, **73**, 2361.

KADIN, M. E. & BENSCH, K. G. (1971) On the origin of Ewing's tumour. *Cancer.*, **27**, 257.

MADRI, J. A., DREYER, B., PITLICK, F. A. & FURTHMAYR, H. (1980) The collagenous components of the subendothelium: correlation of structure and function. *Lab. Invest*, **43**, 403.

PROCKOP, D. J., KIVIRIKKO, K. I., TUDERMAN, L. & GUZMAN, N. A. (1979) The biosynthesis of collagen and its disorders (second of two parts) *N. Engl. J. Med.*, **30**, 77.

REMBERGER, K. & GAY, S. (1977) Immunohistochemical demonstration of different collagen types in the normal epiphyseal plate and in benign and malignant tumours of bone and cartilage. *Z. Krebsforsch.*, **90**, 95.

ROESSNER, A., VOSS, B., RAUTERBERG, J. & IMMENKAMP, M. (1980) Ewing's sarcoma. A comparative electron and immunofluorescence microscopical study. *13th International Congress of the International Academy of Pathology*. Abstract. Paris: International Academy of Pathol. p. 159.

STERN, R., WILCZEK, J., THORPE, W. P., ROSENBERG, S. A. & CANNON, G. (1980) Procollagens as markers for the cell or origin of human bone tumours. *Cancer Res.*, **40**, 325.

STOUT, A. P. (1943) Hemangio-endothelioma: a tumour of blood vessels featuring vascular endothelial cells. *Ann. Surg.*, **118**, 445.

SYKES, B., PUDDLE, B., FRANCIS, M. & SMITH, R. (1976) Estimation of two collagens in human dermis by interrupted gel electrophoresis. *Biochem. Biophys. Res., Commun.* **72**, 1472.

WEBSTER, D. F. & HARVEY, W. (1979) A quantitative assay for collagen synthesis in microwell fibroblast cultures. *Analyt. Biochem.*, **96**, 220.